# A Guide to Basic Statistics for Educational Research

**DOI:** 10.15766/mep_2374-8265.11187

**Published:** 2021-10-04

**Authors:** Donna M. Windish

**Affiliations:** 1 Associate Professor of General Internal Medicine, Department of Internal Medicine, Yale School of Medicine; Director, Resident Research, Yale Primary Care Residency Program, Yale School of Medicine; Program Director, General Internal Medicine Medical Education Fellowship, Yale School of Medicine; Director, Advancement of Clinician-Educator Scholarship (ACES) Faculty Development Program, Department of Internal Medicine, Yale School of Medicine

**Keywords:** Statistics, Faculty Development, Case-Based Learning, Quantitative Research

## Abstract

**Introduction:**

Clinician-educators often need to produce scholarship for academic promotion. While some programs exist to help with faculty development skills, few provide adequate statistical training to help educators evaluate their work.

**Methods:**

From January 2020 through January 2021, faculty at three academic centers attended one of five in-person or virtual seminars with dedicated statistical training for medical education interventions. These 90-minute seminars included a 45-minute PowerPoint presentation of common statistical tests used for educational interventions followed by small breakout groups to help attendees work on additional practice examples. After each seminar, surveys were distributed in person or virtually to obtain feedback.

**Results:**

Forty-three faculty attended the five seminars, with a range of surgical and nonsurgical specialties represented. Of these attendees, 38 (88%) completed session evaluations. The majority of respondents (*n* = 34, 90%) rated the session as extremely useful in helping them know how to use statistics in their scholarly work. Most participants agreed or strongly agreed they had adequate time to practice skills (*n* = 30, 79%). Self-rated confidence in using statistics was significantly higher after the session compared to before (3.00 post vs. 1.97 pre, *p* < .0001). Most participants (*n* = 32, 84%) rated the session as excellent and the small-group practice as most useful (*n* = 16, 42%), but many (*n* = 26, 69%) wanted more skills practice.

**Discussion:**

This intervention shows that dedicated training on biostatistics used in educational interventions can help clinician-educators improve self-rated confidence and knowledge in choosing statistical tests in educational scholarship.

## Educational Objectives

By the end of this activity, learners will be able to:

1. Describe the application of the following statistical areas to educational interventions: study designs, variable types, exploratory data analysis, confirmatory (inferential) data analysis, and basic interpretation of results.
2. Use a four-step approach to choosing a statistical test for educational cases.

## Introduction

Producing scholarship is often a key determinant to academic advancement for clinician-educators regardless of home institution.^1–3^ The value of educational scholarship is well recognized and is receiving increased support.^4^ To help clinician-educators cultivate faculty development skills, some institutions have established academies of medical educators^5^ or education scholarship units.^6^ Despite these initiatives, many institutions may not provide adequate statistical or methodological support to help with development and evaluation of educators' work.^3^

A recent Association of American Medical Colleges survey showed that a majority of medical schools lack specific biostatistics training.^7^ This lack of training can contribute to low statistical knowledge among resident trainees^8–11^ and subsequently to low statistical literacy among faculty.^11–13^ A recent scoping review of clinician-educator faculty development programs found that few programs focus on research or scholarship skills.^14^ Without statistical knowledge, clinician-educators may be at a disadvantage in publishing their scholarly work and thus potentially miss opportunities to be promoted.

Resources exist that address understanding statistical concepts and evidence-based medicine. The *JAMA* Guide to Statistics and Medicine contains a series of articles addressing statistical techniques used in clinical research.^15^ The goal of the series is to help clinicians understand and learn how to critically appraise the medical literature. One article in the series reviews the reporting guidelines for survey studies.^16^ Since survey research is a common tool used in educational interventions, educators might find this particular article helpful in their work. A recent publication in *MedEdPORTAL* describes a module for teaching students basic biostatistics and evidence-based medicine.^17^ The authors of that resource review study design strengths and weaknesses, how to appraise the literature, and how to assess the clinical importance of published studies. Another workshop in *MedEdPORTAL* contains an interactive review of basic biostatistics and discusses how to apply Bayes' theorem to testing and decision-making.^18^ It uses a flipped classroom approach with quizzes to assess knowledge gained. Each of the three publications just described can aid educators in understanding basic statistical concepts, evidence-based medicine, and reading of the literature. None provide a dedicated guide that would aid educators in choosing statistical tests when analyzing their own educational interventions.

In 2006, Windish and Diener-West developed a guide to help clinician-educators understand and choose statistical tests.^19^ Since then, little has been published that provides specific training on statistics for educational interventions with detailed examples. The resource presented here is a unique contribution to the literature aimed at building knowledge of biostatistics using educational examples that clinician-educators will find germane to their educational scholarship. The resource includes an instructional video identical to content presented in faculty development seminars across multiple institutions taught to medical educators. It also provides active learning opportunities through additional educational examples to practice and apply what has been learned from the video. This resource can be used as a seminar at other institutions in addition to serving as an everlasting resource for individuals when conducting educational research.

## Methods

Five faculty development seminars were offered at three different schools of medicine from January 2020 through January 2021. Seminars were either in person or virtual via Zoom, with a range of six to 12 participants, and lasted 90 minutes. Each seminar was led by the author and included a 45-minute PowerPoint presentation that reviewed study designs, variable types, exploratory data analysis, confirmatory data analysis, basic interpretation of results, and a four-step approach to choosing a statistical test.^19^ Statistical content was determined based on the low literacy regarding these concepts seen in prior studies of residents and educators.^8–13^ A video of the PowerPoint presentation contained in this seminar is available in Appendix A. All figures in the presentation were created by the author using Stata statistical software version 14.2 (StataCorp) from fabricated data for illustrative purposes only. The photographs in the apple-pie analogy for regression analysis are author owned. In each seminar, statistical concepts were introduced and interwoven throughout the presentation using an example of an educational intervention aimed at improving second-year medical students' counseling skills, confidence in medical interviewing, professionalism skills, and pass rate. This example was designed to address how to evaluate a curriculum using different evaluation strategies, including the broad categories of assessing knowledge, attitudes, and skills. Statistical concepts included continuous, ordinal, and dichotomous outcome variables, parametric tests, nonparametric tests, and paired analyses.

After the PowerPoint presentation, faculty divided into smaller groups of two to five people who worked together for 20 minutes on additional practice examples provided on worksheets (Appendix B). This small-group practice allowed participants to apply the statistical knowledge learned in the presentation. All figures in the worksheets were created by the author from manufactured data and used for illustrative purposes. Half of the small groups completed questions from case 1, and the other half completed questions from case 2. Case 1 addressed the following statistical concepts: Student *t* test, correlation, and multiple logistic regression. Case 2 had participants work through examples that used a paired *t* test and analysis of variance. The last 15 minutes of the seminar featured a debrief of the practice examples with answers provided in the larger group (Appendix C).

Seminars were held at the Yale School of Medicine, the Washington University School of Medicine in St. Louis, and the University of Wisconsin School of Medicine and Public Health. The two sessions held at Yale were hosted by the Department of Medicine and were open to all faculty in the department, with one in-person session and one virtual session. The two in-person sessions held at the Washington University in St. Louis were hosted by the Academy of Educators and were open to all faculty in any discipline throughout the university. One seminar was hosted virtually for the University of Wisconsin and was open to educators in graduate and undergraduate medical education.

At the end of each seminar, faculty were asked to complete a session evaluation (Appendix D). Questions asked participants to rate the following:

1. The usefulness of the session in helping know them how to use statistics in their current scholarly work (5-point scale: 1 = *extremely useful,* 5 = *extremely useless*),
2. The adequacy of the faculty facilitator (5-point scale: 1 = *extremely adequate,* 5 = *extremely inadequate*),
3. The adequacy of time to practice skills (5-point scale: 1 = *strongly disagree,* 5 = *strongly agree*),
4. Their confidence in using statistics before and after the seminar (4-point scale: 1 = *very unconfident,* 4 = *very confident*), and
5. The overall session (5-point scale: 1 = *poor,* 5 = *excellent*).

Open-ended questions were also asked to elucidate the most useful part of the session and recommendations for change. A paired *t* test was used to compare self-rated confidence before and after the session.

Appendix E is a facilitator guide providing a step-by-step approach to replicating the previously described seminar. It features information on how to develop a successful session, including what to do prior to the session, how to use the video in Appendix A, how to execute the small-group breakout session, and how to review the answers to the small-group work. The video can be stopped at various times to discuss each area covered. Educational and statistical topics with corresponding video start times are as follows:

• Start time: 0:03:02—educational example question 1: counseling skills.
• Start time: 0:03:25—study designs.
• Start time: 0:04:43—paired data versus unpaired data.
• Start time: 0:07:40—types of outcome research variables.
• Start time: 0:11:31—exploratory data analysis.
• Start time: 0:14:27—parametric tests.
• Start time: 0:16:06—nonparametric tests.
• Start time: 0:17:55—confirmatory data analysis.
• Start time: 0:18:46—regression analysis.
• Start time: 0:25:34—hypothesis testing.
• Start time: 0:29:30—educational example question 2: confidence in skills.
• Start time: 0:32:33—educational example question 3: professionalism skills.
• Start time: 0:35:25—educational example question 4: pass rate.
• Start time: 0:36:01—dichotomous outcomes.

## Results

In total, 43 faculty attended the five sessions, with a range of surgical and nonsurgical specialties represented, including general surgery, orthopedics, radiology, internal medicine, nephrology, pulmonology, cardiology, geriatrics, gastroenterology, ophthalmology, pediatrics, physical therapy, genomics, psychiatry, pathology, and dermatology. Of the 43 faculty, 38 (88%) completed session evaluations. Most respondents (*n* = 34, 90%) rated the session as extremely useful in helping them know how to use statistics in their current scholarly work. All 38 respondents (100%) rated the facilitator as extremely adequate at teaching the statistical concepts. Most participants agreed or strongly agreed that they had adequate time to practice skills in the small-group breakout session (*n* = 30, 79%). Self-rated confidence in using statistics was significantly higher after the session compared to before (3.00 post vs. 1.97 pre, *p* < .0001). Most participants (*n* = 32, 84%) rated the session as excellent, with the remainder rating it as very good (*n* = 6, 16%).

All respondents provided at least one comment on the most useful part of the session. The majority of comments listed the small-group practice as most useful (*n* = 16, 42% of all comments), followed by the usefulness of the flowcharts to determine which statistical test to use (*n* = 14, 36%). Other faculty felt the session helped demystify statistics (*n* = 7, 18%) or found the descriptions of when to use statistical tests most useful (*n* = 2, 5%).

Thirty-two faculty (84%) provided recommendations for change. Having more examples (*n* = 12, 38%) and more time for practice (*n* = 10, 31%) were the top two recommendations. Remaining suggestions for change included providing an opportunity for hands-on practice with statistical software (*n* = 4, 12%) and having a specific slide that included a link to the Windish and Diener-West reference^19^ (*n* = 3, 9%).

## Discussion

This educational seminar shows that dedicated training on statistics using educational interventions can provide guidance to clinician-educators in conducting and analyzing their work. The seminars were well received by faculty across a spectrum of specialties, disciplines, and institutions, with universal agreement regarding the sessions' usefulness in helping participants in their educational scholarly work. The versatility of the seminars was also demonstrated as they were done both in person and virtually.

In thinking about lessons learned, I realize that although the educational examples worksheet (Appendix B) allows participants to practice with additional examples, some faculty may wish to practice and obtain feedback using their own scholarship or might want a review of how to employ statistical software. I initially contemplated having such activities as part of the seminar but feel that participants need time to digest the material and think about how to apply what they have learned to their own work. Consequently, to help solidify the concepts, in the future I will be offering an optional follow-up 90-minute session a few weeks after each seminar. One goal of this additional session will be to provide feedback to any participant who wants to demonstrate how they have used the statistical approach taught with their own educational scholarship. Faculty will be asked to use the last page of the educational examples worksheet to guide them in their own initiatives and in presenting their thought process. Another goal of this follow-up session will be to review how to utilize SPSS and Microsoft Excel for statistical analyses using participant data. I have chosen these two platforms as they contain easy-to-use analytic software. If faculty do not have their own data to analyze, I will provide a small sample educational database for them to work through.

Certain limitations to this method of training should be considered. First, the detailed video may not answer all questions that viewers have on statistics despite multiple examples and practice. While the video is comprehensive and covers many statistical tests that clinician-educators can use, it does not cover all possible statistical tests, qualitative assessment, curriculum development, or how to choose evaluation instruments. Some of these limitations can be offset by using Appendix 1 in the Windish and Diener-West article^19^ and textbooks that address curriculum development^20^ and educational research.^21^ In addition, some of the original wording of the evaluation tool may not have captured all participants' opinions. Thus, changes to the session evaluation (Appendix D) have been made. Question 2 now reads, “How would you rate the Statistics facilitator in presenting the content materials covered?” Question 4 now asks, “Was there a part of the Statistics session that was most useful for you? If so, please describe what and why.” This is in place of asking what part of the seminar the participant felt was most useful. Questions 6 and 7, which rate participant confidence, now contain a neutral category to be consistent with other questions featuring 5-point Likert-scale responses. Finally, given the immediate-post design of the session evaluation, it is unclear if the seminars foster actual long-term improvement in subsequent clinician-educator work.

The video and educational examples provided here can be used as a lasting reference for dedicated teaching and practice of evaluating educational initiatives. This resource can be used by institutions that need ways to help their faculty in their educational scholarship pursuits or by individuals who need a guide in analyzing their work. With more faculty reviewing these materials and using them in their educational work, more long-term outcomes can be assessed.

## Appendices


Guide to Basic Statistics for Educational Research.mp4Educational Examples Worksheet.docEducational Examples Answer Sheet.docSession Evaluation.docxFacilitator Guide.docx
*All appendices are peer reviewed as integral parts of the Original Publication.*
