# Supplementary material for: A Guide to Basic Statistics for Educational Research
Source: MedEdPORTAL. 2021 Oct 4;17:11187. doi: 10.15766/mep_2374-8265.11187 (PMC8488064; doi:10.15766/mep_2374-8265.11187)
Supplement: Supplementary file 1 — Guide to Basic Statistics for Educational Research.mp4Educational Examples Worksheet.docEducational Examples Answer Sheet.docSession Evaluation.docxFacilitator Guide.docx [file mep_2374-8265.11187-s001.zip › D. Session Evaluation.docx]

**A Guide to Basic Statistics for Educational Research**

**Session Evaluation**

1. How useful was the Statistics session in helping you to understand how statistics may be used in your current scholarly project?

- Extremely useful
- Moderately useful
- Neither useful nor useless
- Moderately useless
- Extremely useless

2. How would you rate the Statistics facilitator in presenting the content materials covered?

- Extremely adequate
- Moderately adequate
- Neither adequate nor inadequate
- Moderately inadequate
- Extremely inadequate

3. The Statistics session gave me enough time to practice skills learned with hands on activities.

- Strongly disagree
- Disagree
- Neither agree nor disagree
- Agree
- Strongly agree

4. Was there a part of the Statistics’ session that was most useful for you? If so, please describe what and why.

5. What recommendations for change do you have for the Statistics session?

6. How would you rate your confidence in using statistics **before** this Statistics session?

- Very Unconfident
- Somewhat Unconfident
- Neither Confident or Unconfident
- Somewhat Confident
- Very Confident

7. How would you rate your confidence in using statistics now **after** the session?

- Very Unconfident
- Somewhat Unconfident
- Neither Confident or Unconfident
- Somewhat Confident
- Very Confident

8. Overall, please rate the Statistics session.

- Poor
- Fair
- Good
- Very Good
- Excellent
