# Supplementary material for: A Guide to Basic Statistics for Educational Research
Source: MedEdPORTAL. 2021 Oct 4;17:11187. doi: 10.15766/mep_2374-8265.11187 (PMC8488064; doi:10.15766/mep_2374-8265.11187)
Supplement: Supplementary file 1 — Guide to Basic Statistics for Educational Research.mp4Educational Examples Worksheet.docEducational Examples Answer Sheet.docSession Evaluation.docxFacilitator Guide.docx [file mep_2374-8265.11187-s001.zip › E. Facilitator Guide.docx]

**Guide to Implementing A Statistics for Medical Educators Seminar**

**1. Developing a successful session**

a. The session can be run in 90 minutes. This will include watching the video, working in small groups on the worksheet questions, reviewing small group answers and having time for questions. NOTE: You can add an additional 15 minutes of time to the session and have participants work through the 4-step approach to choosing statistical tests using examples from their current work. If you do this, have participants use page 7 of the worksheet for individual work and then at the end of the session, ask for 1-2 volunteers to walk through their specific educational question and the 4-step approach they used.

b. If you feel uncomfortable with the content when first starting to run these sessions, consider finding a person at your institution with more statistical experience (not necessarily on the education faculty) to either run or co-run the session. This person can provide expertise and be helpful in answering questions that may be more involved. In time, having one or more educational faculty as an expert in the session content will be helpful to provide an educator’s perspective on statistics and evaluation and to help facilitate the small groups.

c. Aim to recruit 8-12 participants per session. This will allow everyone to have an opportunity to ask questions while keeping the session going.

d. Most successful sessions have participants with active projects, but all faculty can benefit from the session, even if it may serve as a review to some.

e. Sessions can either be run in person or virtually. If doing a virtual session, be sure to have a platform that allows participants to join breakout groups for the worksheet exercises. If in person, be sure there is enough space in the room to have smaller breakout groups work in different areas of the room.

**2. Prior to the session**

a. Assign the Windish/Diener-West^1^ article for reading prior to the session. Send this out about 1 week before the session to allow participants time to read and understand the material.

b. Have the flow diagrams in Appendix 1 of the Windish/Diener-West article available to all participants either in paper form or electronically to be used during the session.

**3. Starting the session: Time 5 minutes**

a. Before starting the video, ask the group if they had a chance to review the Windish/Diener-West article and see if there are any questions from the reading. In particular, ask about any questions concerning how to use the flow diagrams in Appendix 1.

b. Ask participants their comfort in understanding and using statistics in their educational work. This will give you a sense of who your audience is and their statistical comfort.

^1^ **Windish DM**, Diener-West M. A clinician-educator’s roadmap to choosing and interpreting statistical tests. *J Gen Intern Med.* 2006;21:656-60*.*

**4. Using the video recording: Time 45-50 minutes**

a. Start the video. There are natural breaks in the video to pause and ask questions of the group. For each of the 4 research questions posed for the sample 2^nd^-year medical student curriculum in the video (counseling skills, confidence in medical interviewing, professionalism skills and pass rate), pause the video before answers are given at:

i. *Each Step 2 that asks what the study design is*. Remind the group about the difference
 between observational vs. experimental designs.

ii. *Each question that asks about paired or unpaired data*.

iii. *Each Step 3 that asks what the type of outcome variable is*. If there is an ordinal variable, remind participants that sometimes ordinal variables can be considered continuous if there are 5 or more points on the scale.

iv. *Each Step 4 that asks what the distribution of the outcome variable is*. If there is a question about parametric vs. nonparametric distributions just by looking at the plot, you can suggest to the group that they carry out both types of tests and see if they provide similar answers. If answers are similar, use the parametric test.

For each stopping point above, ask the group to tell you what they think the answer is and why. You can do this with a polling software, like Poll Everywhere, to encourage full group participation. Alternatively, you can do this as a “shout out” if in person or in the chat format in an online platform if virtual.

b. During the section on paired analyses, make a point of saying that in pre-post samples, you need to know who each participant is in the study before and after the intervention. You can do this by having each of the targeted learners provide their names in all assessments or by setting up a unique code for each participant to use for each assessment. An example of how to set up a unique code: have targeted learners use the year of their birth and the first 3 letters of their mother’s first name for each assessment.

c. At the end of the video, ask for any other questions that weren’t addressed or need clarification.

**5. Breaking into small groups and using the practice worksheets: Time 15-20 minutes**

a. You can pause here for a short break or continue to the small group work.

b. Be sure each person has Appendix 1 from the Windish/Diener West article available for this part of the session. Remind participants to use the flow sheets to determine what statistical test to use for each null hypothesis question.

c. Divide participants into even numbers of groups. Ideally, there should be 3-4 people in each small group to allow for maximum participation. You can randomly assign participants to groups. If there are faculty with higher levels of statistical knowledge attending, you can consider placing one of these faculty in each of the groups as they could help guide the others through the questions.

d. Assign numbers to each group. Have the odd numbered groups complete the questions together for Case 1. Have the even numbered groups complete the questions together for Case 2. Ask them to answer each question step by step for each Null Hypothesis.

e. Ask the small groups to assign one person as a note keeper. This person should prepare answers for the larger group discussion.

f. If there is only one facilitator for the seminar, that person can move from group to group to check in on the worksheet progress and answer any questions. Have the facilitator stay for the entirety of at least one of the Null Hypothesis questions to see the group’s thought process. If there is more than one facilitator, consider assigning one facilitator to one or more groups.

g. Before the small groups start, remind them that the worksheets will cover similar topics to the video to help solidify concepts, but will also provide additional content including regression analysis, ANOVA and correlation.

NOTE: normality may be questionable for some of the figures. Remind the group that if there is a question about normality, try both the parametric and nonparametric tests to see if answers are similar. If so, they can use the parametric test.

h. Helpful tips for Case 1. While you are visiting group(s) doing Case 1:

i. For Null Hypothesis 1, there might be confusion about using a historical control. This is just an experimental study using a different (historical) control group.

ii. For Null Hypothesis 2, correlation does not have an outcome. Discuss with the group that if both plots of continuous outcomes are normally distributed, use Pearson’s correlation. If either or both plots are nonparametric, you need to use Spearman rank correlation.

i. Helpful tips for Case 2. While you are visiting group(s) doing Case 2:

For Null Hypothesis 1, normality may be in question for all four plots, but for this case, the distributions were considered close enough to be normal, so a parametric test was used.

j. If any group finishes their questions prior to the end of the 20 minutes, that group can start working on the other case for the remainder of the time.

**6. Regrouping to the large group to review worksheet answers: Time 10 minutes**

a. Once the larger group reconvenes, do a quick check-in to see how the group work went for everyone.

b. Have the answer sheet available for your reference so you can provide accurate answers to the questions.

c. Have the/one volunteer note keeper for **Case 1** present the answers for the questions in their case. Ask them to answer each question step by step for each Null Hypothesis. Have them name the flow diagram they used for the 4-step process. Allow the other group(s) who did not work on Case 1 to ask questions. Provide any clarification as needed for the answers.

d. Repeat the process above for **Case 2**.

e. Once all cases are reviewed together, pass out or email the worksheet answers to the participants for them to keep and review.

**7. End of the session: Time 5 minutes**

a. Allow a few minutes to debrief the session with the participants. Ask if there are remaining concepts that are still unclear. Remind participants that not all statistical concepts are covered in the session and that they should/could consider asking a statistician for help with their evaluation if they are confused.

b. Distribute the feedback form for participants to complete.

**8. Consider a follow-up in a few weeks**

Consider a brief follow up with the group by email. Use that time to see if anyone attempted to use the 4-step approach in their own work. Have participants share any successes or challenges in using what they learned.
