# Supplementary material for: A Guide to Basic Statistics for Educational Research
Source: MedEdPORTAL. 2021 Oct 4;17:11187. doi: 10.15766/mep_2374-8265.11187 (PMC8488064; doi:10.15766/mep_2374-8265.11187)
Supplement: Supplementary file 1 — Guide to Basic Statistics for Educational Research.mp4Educational Examples Worksheet.docEducational Examples Answer Sheet.docSession Evaluation.docxFacilitator Guide.docx [file mep_2374-8265.11187-s001.zip › B. Educational Examples Worksheet.doc]

**CASE 1**

A. **Case**: Wellness curriculum for internal medicine residents
B. **Study Aim(s)** – An intervention for residents aimed at improving wellness

C. **Study Design**: Experimental with historical control

60 residents	60 residents

Historical control

Wellness intervention

________________________________________________________________________

**Null Hypothesis 1**: Mean number of days spent taking time out for oneself will not differ between the two groups.

4 Step Approach:

1. Step 1 – List the Study Question
2. Step 2 – List the Study Design
3. Step 3 – List the Type of Outcome Variable
4. Step 4 – List the Distribution of the Outcome Variable given the histograms below:

Intervention days

Control Days

5. What statistical test would you choose?
6. Results: Mean number of days taking time out for oneself (95% CI)

Intervention Group: 223.1 (207.3 – 238.9) days

Historical Group: 156.8 (147.7 – 165.9) days

p <0.001

7. Please describe your interpretation of the results?

**CASE 1 continued**

**Null Hypothesis 2:** No correlation exists between mean duration of burnout measured in weeks and mean sleep duration measured in hours.

4 Step Approach:

1. Step 1 – List the Study Question
2. Step 2 – List the Study Design
3. Step 3 – List the Type of Variables. (NOTE: in this case, there is no true outcome variable. However, you should still understand each type of variable used to make comparisons).
4. Step 4 – List the Distribution of the Variables given the figures below:

Burnout wee

ks

Sleep duration, hours

5. What statistical test would you choose?
6. Results: The correlation between the two variables is: r = 0.034, p = 0.71

Note:	r = 0; no correlation

r = 1; perfect positive correlation

r = -1; perfect negative correlation

r = 0.034,
p = 0.71

Burnout wee

ks

Sleep duration, hours

7. What is your interpretation of these results?

**CASE 1 continued**

**Null Hypothesis 3:** No association exists between the two resident groups and having burnout while controlling for resident age, spirituality, and exercise activity.

4 Step Approach:

1. Step 1 – List the Study Question
2. Step 2 – List the Study Design
3. Step 3 – List the Type of Outcome Variable
4. Step 4 – List the Distribution of the Outcome Variable given the histogram below:

No

Yes

No

Yes

Experiencing Burnout	Experiencing Burnout

5. What statistical test would you choose?
6. Results: Odds Ratio (95% Confidence Interval)

OR = 3.94 (1.50 - 10.38), p=0.006

7. What is your interpretation of these results?

**CASE 2**

**A. Case Description**: National survey study of career satisfaction of clinician-educators.

**B. Study Aim(s):** To determine career satisfaction of clinician-educators.

**C**. **Study Design**: Cross sectional survey

________________________________________________________________________

**Null Hypothesis 1:** There is no difference in age of clinician-educators in four different specialties surveyed: internal medicine, pediatrics, neurology and surgery.

4 Step Approach:

1. Step 1 – List the Study Question
2. Step 2 – List the Study Design
3. Step 3 – List the Type of Outcome Variable
4. Step 4 – List the Distribution of the Outcome Variable given the histograms below:

Internal Medicine	Surgery

Age, years

Age, years

Pediatrics	Neurology

Age, years

Age, years

**CASE 2 continued**

5. What statistical test would you choose?

6. Results:

Mean Age

Internal Medicine 47±12 years

Pediatrics	51±9 years

Surgery 49±10 years

Neurology 49±12 years

p=0.41

7. What is your interpretation of these results?

**CASE 2 continued**

**Null Hypothesis 2:** There is no difference in career satisfaction measured on a scale of -10 (poor satisfaction) to +10 (high satisfaction) comparing faculty before and after making tenure.

4 Step Approach:

1. Step 1 – List the Study Question
2. Step 2 – List the Study Design
3. Step 3 – List the Type of Outcome Variable
4. Step 4 – List the Distribution of the Outcome Variable given the histograms below:

Before Tenure	After Tenure

5. What statistical test would you choose?
6. Results:

Before tenure satisfaction: -2.1% (4.8)

After tenure satisfaction: -3.3% (7.3)

p=0.40

7. What is your interpretation of these results?

**YOUR CASE**

**A. Case Description**:

**B. Study Aim(s):**

**Null Hypothesis:**

4 Step Approach:

1. Step 1 – List the Study Question
2. Step 2 – List the Study Design
3. Step 3 – List the Type of Outcome Variable
4. Step 4 – List the Distribution of the Outcome Variable:
5. What statistical test would you choose?
6. Results:

7. What is your interpretation of these results?
