# Supplementary material for: A Guide to Basic Statistics for Educational Research
Source: MedEdPORTAL. 2021 Oct 4;17:11187. doi: 10.15766/mep_2374-8265.11187 (PMC8488064; doi:10.15766/mep_2374-8265.11187)
Supplement: Supplementary file 1 — Guide to Basic Statistics for Educational Research.mp4Educational Examples Worksheet.docEducational Examples Answer Sheet.docSession Evaluation.docxFacilitator Guide.docx [file mep_2374-8265.11187-s001.zip › C. Educational Examples Answer Sheet.doc]

**CASE 1: Wellness curriculum for internal medicine residents**

**Null Hypothesis 1**: Mean number of days spent taking time out for oneself will not differ between the two groups.

1. **Step 1 – Study Question** Will mean number of days spent taking time out for oneself differ between historical control and intervention groups?

**2. Step 2 – Study Design** Experimental with historical control

3. **Step 3 – Type of Outcome Variable**	Mean days is a continuous variable
4. **Step 4 – Distribution of the Outcome Variable**	Normally distributed
5. **Statistical test**	Student t-test
6. **Results: Mean glucose level (95% Confidence Interval)**

Intervention group: 223.1 (207.3 – 238.9), Historical Group: 156.8 (147.7 – 165.9); p <0.001

**7. Interpretation of the results** Reject the null hypothesis, and conclude: The intervention

group has significantly higher number of days taking care of oneself than the historical group.

**Null Hypothesis 2:** No correlation exists between mean duration of burnout measured in weeks and mean sleep duration measured in hours.

1. **Step 1 – Study Question** Is there a correlation between duration of burnout and sleep?
2. **Step 2 – Study Design** Correlation between two variables.
3. **Step 3 – Type of Variables.** Duration of burnout in weeks: continuous; Mean sleep in hours: continuous

4. **Step 4 – Distribution of the Variables** Duration of sleep: normally distributed,

Duration of burnout: non-normally distributed.

5. **Statistical test**	Spearman rank correlation
6. **Results:** r = 0.034, p = 0.71

7. **Interpretation of results** Accept the null hypothesis and conclude no significant correlation between variables.

**Null Hypothesis 3:** No association exists between the two resident groups and having burnout while controlling for a residents age, spirituality, and exercise activity.

1. **Step 1 – Study Question** Is there an association between the two resident groups while
   controlling for resident age, spirituality, and exercise activity.

**2. Step 2 – Study Design** Pre post study quantifying the association between:

group assignment (independent variable) and burnout (the outcome or dependent

variable) while controlling for age, spirituality and exercise activity (covariates).

**3. Step 3 – Type of Outcome Variable**	Having burnout is dichotomous (yes/no)

***Case 1 Continued***

**4. Step 4 – Distribution of Outcome Variable**	Binomial distribution

5. **Statistical test**	Multiple logistic regression
6. **Results:**	The Odds Ratio is: OR = 3.94 (95% CI = 1.50 – 10.38); p = 0.006

**7. Interpretation of results** Reject the null hypothesis, and conclude: There is a 3.94 times

increased odds of having burnout in the historical group compared to the intervention group while controlling for age, spirituality and exercise activity.

**CASE 2: National survey study of career satisfaction of clinician-educators**

**Null Hypothesis 1:** National survey study of career satisfaction of clinician-educators.

1. **Step 1 – Study Question** Is there a difference in age of clinician-educators in four different specialties surveyed: internal medicine, pediatrics, neurology and surgery.
2. **Step 2 – Study Design**	Survey study
3. **Step 3 – Type of Outcome Variable**	Age is continuous
4. **Step 4 – Distribution of the Outcome Variable** All are normally distributed
5. **Statistical test** Analysis of Variance to compare means
6. **Results:** Internal Medicine 47±12 yrs; Surgery 51±9 yrs; Pediatrics 49±10 yrs; Neurology 49±12 yrs, p=0.41

**7. Interpretation of results** Cannot reject the null hypothesis and conclude that there is no

difference in age between groups.

**Null Hypothesis 2:** There is no difference in career satisfaction measured on a scale of -10 (poor satisfaction) to +10 (high satisfaction) comparing faculty before and after making tenure.

1. **Step 1 – Study Question** Is there a difference in career satisfaction before and after tenure?
2. **Step 2 – Study Design**	Cross sectional survey with a pre-post design
3. **Step 3 – Type of Outcome Variable**	Satisfaction is a continuous variable
4. **Step 4 – Distribution of the Outcome Variable** Both pre-post are normally distributed

5. **Statistical test**	Paired t-test
6. **Results:** Before tenure satisfaction: -2.1 (4.8); After tenure satisfaction: -3.3 (7.3), p=0.40

**7. Interpretation of results** Cannot reject the null hypothesis and conclude that there is no

difference in career satisfaction before and after tenure.
